# The burden of blast injuries on a level i trauma center: a comparative analysis of blast versus blunt trauma

**DOI:** 10.1007/s00068-026-03356-5

**Published:** 2026-09-28

**Authors:** Elchanan Quint, Guy Barsky, Anton Osyntsov, Ilya Replyanski, Gad Shaked

**Affiliations:** 1 Department of General Surgery B, Soroka University Medical Center, Beer Sheva, Israel; 2 Faculty of Health Sciences, Ben-Gurion University of the Negev, Beer Sheva, Israel; 3 Trauma Unit, Soroka University Medical Center, Beer Sheva, Israel

**Keywords:** Blast injury, Blunt trauma, Trauma center, Resource utilization, Injury severity score, Mass casualty

## Abstract

**Purpose:**

We hypothesized that blast trauma results in a greater institutional resource burden than blunt trauma.

**Methods:**

This retrospective study compared blast trauma patients (N = 321) to blunt trauma patients (N = 9,172). A Propensity Score Matching (PSM) on Age, Sex and Injury Severity Score (ISS) was executed, yielding 1,481 matched blunt controls. The primary composite endpoint was Institutional Resource Burden, defined as requiring one or more of the following high-intensity resource metrics: prolonged hospital length of stay (LOS >7 days), ICU admission, or need for urgent surgical intervention (<6 hours from admission). Secondary endpoints included in-hospital mortality, shock-trauma room triage, any surgical intervention, multiple operations (≥ 3), massive transfusion protocol (MTP) activation, requirement for mechanical ventilation, and ICU/hospital LOS.

**Results:**

Blast patients demonstrated a higher rate of the primary composite institutional burden (78.2 vs. 51.2, OR = 3.42, 95% CI: 2.59 - 4.51, p <0.0001). Blast mechanism independently predicted shock-trauma room triage (OR = 10.60, 95% CI: 8.18 - 13.75, p <0.0001), urgent surgery (OR = 7.49, 95% CI: 5.73 - 9.80, p <0.0001), ≥3 operations (OR = 12.40, 95% CI: 8.59 - 17.90, p <0.0001), MTP activation (OR = 11.68, 95% CI: 6.48 - 21.05, p <0.0001), and ICU admission (OR = 5.54, 95% CI: 4.37 - 7.04, p <0.0001). These differences persisted across both ISS <16 and ISS ≥16 strata.

**Conclusion:**

Blast trauma imposes a significantly higher institutional resource burden than blunt trauma across all injury severity strata.

## Introduction

Blast injuries represent a distinct and increasingly relevant challenge for modern trauma systems. Historically confined to battlefield settings, explosive injuries have emerged as a growing concern for civilian trauma centers, as global terrorism, criminal violence, and industrial accidents continue to threaten public safety. While blast injuries remain relatively uncommon in civilian centers - accounting for approximately 0.2–0.3% of admissions in Western countries - they carry significant morbidity and require diagnostic and therapeutic approaches that differ fundamentally from those applied to conventional blunt or penetrating trauma [1, 2].

The pathophysiology of blast injury is multifaceted. Explosions produce complex injury patterns combining primary blast effects from overpressure waves (barotrauma to air-filled organs including the lungs, gastrointestinal tract, and tympanic membranes), secondary injuries from projectile fragments, tertiary injuries from bodily displacement, and quaternary effects including burns and inhalation injury [3]. These mechanisms may act simultaneously or in sequence, and their clinical consequences may evolve over hours to days, complicating early management. In civilian settings, the most common blast etiologies include fireworks detonations (29%), gas or pressurized container explosions (27%), and industrial accidents; combat-related blast injuries are characterized by higher severity, multisystem involvement, and devastating extremity injuries including traumatic amputations [1–4].

Understanding the specific resource requirements of blast trauma is essential for Level I trauma centers that may be called upon to manage both civilian and combat-related explosive injuries. Unlike military trauma systems, which have developed protocols based on extensive combat experience, civilian centers must maintain preparedness for relatively rare but potentially catastrophic events while balancing everyday clinical demands [5]. Our institution occupies a unique position as a civilian Level I trauma center that also receives combat-related blast casualties as the first post-scene tier, resulting in a 10-fold higher proportion of blast admissions (3.4%) compared to typical Western civilian centers [1].

We hypothesized that the clinical and pathophysiological complexity of blast trauma results in substantially greater institutional burden compared to blunt trauma. The purpose of this study was to quantify this burden across multiple resource utilization metrics, and to determine whether increased demands persist across different injury severity strata - including among patients with ISS < 16. The goal is to provide evidence-based insights to support trauma center preparedness, protocol development, and training programs for this challenging patient population.

## Methods

### Study population

A retrospective cohort study was conducted at a certified regional Level I trauma center evaluating all acute trauma admissions between 2023 and 2025 (*N* = 9,493). The study included patients across all age spectrums (0–99 years). Exclusion criteria were limited to patients declared Dead on Arrival (DOA) without resuscitative efforts and records with incomplete registry demography.

### Blast trauma case definition

Blast trauma was defined as any casualty resulting from an explosive detonation, encompassing primary blast overpressure, secondary effect (penetrating injury from fragments), tertiary effect (blunt trauma), or quaternary blast effects (i.e. thermal burns or toxic gas inhalation). Patients were assigned to the Blast group if their primary mechanism of injury was explosion-related, regardless of whether the presentation was a solitary explosion effect or a complex multi-mechanism injury.

### Prehospital triage & resuscitation workflow

Prehospital triage is conducted by regional Emergency Medical Services (EMS) paramedics using standardized protocols based on physiological and anatomical findings and on mechanism of injury. Military casualties occurring in the battlefield which is about 50 km away from our center were treated initially by military paramedics and or physicians who also triaged them. Most severely injured patients were transferred by combined ground transport and helicopters. In multiple casualty incidents all patients arrive to the entrance of the emergency department where a triage physician rapidly sorts them by directing critically injured to shock-trauma room, severely injured to the surgical emergency department, and walking, lightly injured patients to a seperate section in the emergency department.

### Study endpoints & clinical definitions

Primary Composite Endpoint (Institutional Burden): Defined as requiring one or more of the following high-intensity resource utilization metrics: extended total hospital stay (LOS > 7 days), ICU admission, or urgent surgical intervention (< 6 h from admission).

Secondary Outcomes: In-hospital mortality, shock-trauma room triage, surgical intervention, multiple reoperations (≥ 3), massive transfusion protocol (MTP) activation, ICU LOS, required mechanical ventilation, and total ventilator days.

- Urgent vs. Overall Surgery: “Surgery” encompassed any therapeutic operating room procedure performed during index admission. “Urgent Surgery” was defined specifically as any life- or limb-saving surgical procedure initiated within 6 h of emergency department arrival (e.g., emergency laparotomy, thoracotomy, craniotomy, vascular repair, or management of limb amputation or near amputation)
- Multiple operations (≥ 3): Defined as requiring 3 or more distinct operating room sessions during index admission, capturing complex multi-stage damage control interventions, serial wound debridement, multi- disciplinary teams operations, and planned or unplanned re-explorations (as opposed to standard two-stage procedures)
- Probability of Survival Calculation: Predicted survival probability was calculated using the Trauma and Injury Severity Score (TRISS) model, integrating Age, ISS, and the Revised Trauma Score (RTS) upon arrival. This data is created automatically by the trauma registry software

### Statistical analysis & propensity score matching

Continuous variables were tested for normality using the Shapiro-Wilk test and are presented as Mean ± Standard Deviation (SD) as well as Median and Interquartile Range (IQR [25th − 75th percentiles]). Categorical variables are presented as counts and percentages (n, %). Unadjusted comparisons utilized Student’s t-test / Welch’s t-test for continuous data, Mann-Whitney U test for non-normally distributed continuous variables, and Chi-square or Fisher’s exact test for categorical variables.

To account for sample size disparities (*N* = 321 Blast vs. *N* = 9,172 Blunt) and potential baseline confounding, a 1:10 Nearest-Neighbor Propensity Score Matching (PSM) without replacement was executed based on Age, Sex and ISS (caliper 0.02). Additionally, a multivariable logistic regression model was constructed adjusting for Age, Sex, and ISS to calculate Adjusted Odds Ratios (aOR) with 95% Confidence Intervals (95% CI). Subgroup analyses were stratified by injury severity score (ISS < 16 vs. ISS ≥ 16). All statistical analyses were performed using Python 3.11 (SciPy and Statsmodels libraries), with *p* < 0.05 considered statistically significant.

## Results

### Demographics & baseline characteristics

A total of 9,493 patients were evaluated [321 (3.4%) Blast, 9,172 (96.6%) Blunt]. Within the blast cohort, 246 (76.6%) had ISS < 16 and 75 (23.4%) had ISS ≥ 16; within the blunt cohort, 7,959 (86.8%) had ISS < 16 and 1,209 (13.2%) had ISS ≥ 16. Blast patients were significantly younger (25.26 ± 8.82 vs. 39.28 ± 29.31 years, Median: 22.0 vs. 32.0, *p* < 0.0001) and predominantly male (97.2% vs. 62.3%, *p* < 0.0001). Mean ISS was higher in the Blast group (10.16 ± 10.30 vs. 8.30 ± 6.69, Median: 6.0 vs. 9.0, *p* < 0.0001). Following 1:10 PSM, baseline age and ISS imbalances were successfully eliminated (*n* = 321 Blast vs. *n* = 1,481 matched Blunt controls; Age: 25.26 ± 8.82 vs. 26.80 ± 16.37 years, *p* = 0.1820; ISS: 10.16 ± 10.30 vs. 11.26 ± 9.44, *p* = 0.4105). Male predominance remained following the PSM: 312 / 9 (97.2% / 2.8%) vs. 1,214 / 267 (82.0% / 18.0%), *p* < 0.0001 (Table 1).

### Anatomical injury pattern

Anatomical injury distribution by Abbreviated Injury Score (AIS) ≥ 3 revealed distinct patterns between groups. The incidence of severe abdominal injury was not significantly different between the blast and blunt groups (*p* = 0.1900 in the unmatched analysis and *p* = 0.6080 in the PSM analysis). Severe head or thoracic injury were significantly lower in blast (head 17.8% vs. 33.0%, *p* < 0.0001, thoracic 8.7% vs. 16.4, *p* = 0.0002). However, following PSM no significant differences were found (severe head injury 17.8% vs. 22%, *p* = 0.0981, severe thoracic injury 8.7% vs. 12.5%, *p* = 0.0620. The incidence of severe extremity trauma was lower in both the unmatched and matched analyses: 43.3% vs. 81.3%, *p* < 0.0001 and 43.3% vs. 62.0%, *p* < 0.0001, respectively).

**Table 1:** Demographic, clinical, and anatomical injury characteristics

Characteristic	Blast Cohort (*n* = 321)	Blunt Cohort (*n* = 9,172)	Unmatched *p*-value	Matched Blunt Controls (*n* = 1,481)	Matched *p*-value
Male / Female, N (%)	312 / 9 (97.2% / 2.8%)	5,716 / 3,456 (62.3% / 37.7%)	< 0.0001	1,214 / 267 (82.0% / 18.0%)	< 0.0001
Age (years), Mean ± SD [Median, IQR]	25.26 ± 8.82 [22.0, 20.0–28.0]	39.28 ± 29.31 [32.0, 13.0–68.0]	< 0.0001	26.80 ± 16.37 [24.0, 17.0–34.0]	0.1820
ISS, Mean ± SD [Median, IQR]	10.16 ± 10.30 [6.0, 4.0–13.0]	8.30 ± 6.69 [9.0, 4.0–9.0]	< 0.0001	11.26 ± 9.44 [9.0, 4.0–16.0]	0.4105
AIS ≥ 3 Head Injury, N (%)	57 (17.8%)	3,028 (33.0%)	< 0.0001	326 (22.0%)	0.0981
AIS ≥ 3 Thoracic Injury, N (%)	28 (8.7%)	1,507 (16.4%)	0.0002	185 (12.5%)	0.0620
AIS ≥ 3 Abdominal Injury, N (%)	33 (10.0%)	1,170 (13.0%)	0.1900	163 (11.0%)	0.6080
AIS ≥ 3 Extremity Fracture, N (%)	139 (43.3%)	7,455 (81.3%)	< 0.0001	918 (62.0%)	< 0.0001
TRISS Survival Prob (%), Median (IQR)	99.28% (99.01%–99.33%)	99.42% (96.75%–99.61%)	0.0094	99.42% (96.75%–99.61%)	0.0038

### Resource burden & clinical endpoints

In-hospital mortality was low and equivalent between groups (1.2% Blast vs. 1.4% Blunt, OR = 0.89, 95% CI: 0.33–2.43, *p* = 0.8100). However, the primary composite institutional burden was markedly higher in Blast trauma (78.2% vs. 51.2%, OR = 3.42, 95% CI: 2.59–4.51, *p* < 0.0001), and so were the secondary end-points (Table 2). The multivariable logistic regression model that was constructed to adjust for Age, Sex, and ISS to calculate Adjusted Odds Ratios (aOR) with 95% confidence intervals (95% CI) also revealed that blast patients required significantly higher resource burden: shock-trauma room resuscitation (aOR = 9.82, 95% CI: 7.41–13.02, *p* < 0.0001), urgent surgery within 6 h (aOR = 6.84, 95% CI: 5.12–9.14, *p* < 0.0001), ≥ 3 operations (aOR = 10.45, 95% CI: 7.10–15.38, *p* < 0.0001), MTP activation (aOR = 10.12, 95% CI: 5.48–18.68, *p* < 0.0001), ICU admission (aOR = 7.27, 95% CI: 5.21–10.16, *p* < 0.0001), mechanical ventilation (aOR = 12.06, 95% CI: 8.13–17.89, *p* < 0.0001), prolonged hospital stay > 7 days (aOR = 2.98, 95% CI: 2.22–4.01, < 0.0001.

**Table 2:** Primary and secondary resource utilization endpoints (Blast vs. Blunt)

Endpoint	Blast Cohort *n* = 321	Blunt Cohort *n* = 9,172	Odds Ratio (95% CI)	*p*-value
Primary Composite Burden, n (%)	251 (78.2%)	4,696 (51.2%)	3.42 (2.59–4.51)	< 0.0001
In-Hospital Mortality, n (%)	4 (1.2%)	128 (1.4%)	0.89 (0.33–2.43)	0.8100
Shock-Trauma Room Activation, n (%)	243 (75.7%)	2,083 (22.7%)	10.60 (8.18–13.75)	< 0.0001
Overall Need for Surgery, n (%)	136 (42.4%)	4,310 (46.9%)	0.83$ (0.66–1.04)	0.1030
Urgent Surgery (< 6 h from admission), n (%)	83 (25.9%)	408 (4.4%)	7.49 (5.73–9.80)	< 0.0001
Multiple operations (≥ 3), n (%)	44 (13.7%)	116 (1.3%)	12.40 (8.59–17.90)	< 0.0001
MTP Activation, n (%)	16 (4.9%)	41 (0.4%)	11.68 (6.48–21.05)	< 0.0001
ICU Admission, n (%)	115 (35.8%)	839 (9.1%)	5.54 (4.37–7.04)	< 0.0001
Mechanical Ventilation, n (%)	83 (25.9%)	401 (4.4%)	7.63 (5.83–9.98)	< 0.0001
Total Hospital LOS (days), Mean ± SD [Median, IQR]	8.91 **±** 14.83 [3.0, 1.0–9.5]	4.88 **±** 7.32 [3.0, 1.0–5.0]	—	< 0.0001
ICU LOS (days), Mean ± SD [Median, IQR]	10.61 **±** 17.38 [4.0, 2.0–10.0]	5.47 **±** 9.87 [2.0, 1.0–4.0]	—	< 0.0001
Ventilator Duration (days), Mean ± SD [Median, IQR]	11.30 **±** 18.50 [4.0, 2.0–12.0]	4.80 **±** 8.20 [2.0, 1.0–5.0]	—	0.0012

ISS, Injury Severity Score; MTP, massive transfusion protocol; LOS, length of stay; ICU, intensive care unit; OR, odds ratio

Subgroup Analysis by Injury Severity (ISS < 16 vs. ISS ≥ 16).

Subgroup analysis confirmed that the increased resource burden of blast trauma was independent of injury severity (Table 3).

**Table 3:** Comparison of primary and secondary resource utilization endpoints between subgroups of severity of injury (ISS < 16 or ISS ≥ 16 in Blast vs. Blunt groups)

Variable	ISS < 16 Blast (*n* = 246)	ISS < 16 Blunt (*n* = 7,959)	ISS < 16 OR (95% CI)	ISS < 16 *P*	ISS ≥ 16 Blast (*n* = 75)	ISS ≥ 16 Blunt (*n* = 1,209)	ISS ≥ 16 OR (95% CI)	ISS ≥ 16 *P*
Primary Composite Endpoint*	71 (28.9%)	1,377 (17.3%)	1.94 (1.46–2.57)	< 0.0001	70 (93.3%)	815 (67.4%)	6.77 (2.71–16.90)	< 0.0001
Mortality	0 (0.0%)	50 (0.6%)	0.32 (0.02–5.16)	0.212	4 (5.3%)	78 (6.5%)	0.82 (0.29–2.30)	0.701
Shock-Trauma Room	170 (69.1%)	1,338 (16.8%)	11.07 (8.39–14.60)	< 0.0001	73 (97.3%)	745 (61.6%)	22.73 (5.55–93.07)	< 0.0001
Surgery (Any Procedure)	81 (32.9%)	3,786 (47.6%)	0.54 (0.41–0.71)	< 0.0001	55 (73.3%)	523 (43.3%)	3.61 (2.14–6.09)	< 0.0001
Urgent Surgery (< 6 h)	40 (16.3%)	178 (2.2%)	8.49 (5.86–12.29)	< 0.0001	43 (57.3%)	229 (18.9%)	5.75 (3.56–9.29)	< 0.0001
Multiple operations ($\:\ge\:3$)	18 (7.3%)	54 (0.7%)	11.56 (6.67–20.02)	< 0.0001	26 (34.7%)	62 (5.1%)	9.82 (5.72–16.84)	< 0.0001
MTP Activation	3 (1.2%)	8 (0.1%)	12.27 (3.24–46.54)	< 0.0001	13 (17.3%)	32 (2.6%)	7.71 (3.86–15.43)	< 0.0001
ICU Admission	48 (19.5%)	240 (3.0%)	7.80 (5.55–10.96)	< 0.0001	67 (89.3%)	611 (50.5%)	8.20 (3.90–17.21)	< 0.0001
Mechanical Ventilation	22 (8.9%)	68 (0.9%)	11.40 (6.92–18.77)	< 0.0001	61 (81.3%)	333 (27.5%)	11.46 (6.33–20.77)	< 0.0001

*Primary Composite Endpoint includes meeting at least one major acute resource utilization standard: ICU admission, hospital length of stay  days, or urgent surgical intervention ( hours)

Key subgroup insights demonstrated that even among the minor-to-moderate injury cohort $\:\mathrm{of\:}\mathrm{ISS}<16$ blast casualties experienced a nearly double likelihood of meeting the primary composite endpoint ($\:\mathrm{OR}=1.94,\mathrm{CI:\:}1.46\mathrm{\--}2.57$). Blast mechanism remained associated with significant elevations (X 11 the rate) in shock-trauma triage (OR = 11.07, 95% CI: 8.39–14.60), almost X 12 the rate of multiple operations.

(≥ 3) (OR = 11.56, 95% CI: 6.67–20.02), over X 12 the rate of MTP activation (OR = 12.27, 95% CI: 3.24–46.54), and mechanical ventilation at over $\:11\times\:$ the rate ($\:\mathrm{OR}=11.40$) compared to non-blast blunt controls.

The blast trauma patients in the severe injury cohort ($\:\mathrm{ISS}\ge\:16$) demonstrated exceptional resource consumption ($\:93.3\%$ composite rate vs. $\:67.4\%$; $\:\mathrm{OR}=6.77,\mathrm{\:95\%\:CI:\:}2.71\mathrm{\--}16.90$). Shock-trauma triage was (OR = 22.73, 95% CI: 5.55–93.07).

Over one-third required $\:\ge\:3$ multiple surgical procedures (OR = 9.82, 95% CI 5.72–16.84), and $\:81.3\%$ required mechanical ventilation ($\:\mathrm{OR}=11.46,\:95\mathrm{\%}\:\mathrm{C}\mathrm{I}\:$6.33–20.77).

Blast mechanism similarly independently predicted in the severe injury (ISS ≥ 16) sub-group urgent surgery (OR = 5.75, 95% CI: 3.56–9.29), ≥ 3 operations (OR = 9.82, 95% CI: 5.72–16.84), MTP activation (OR = 7.71, 95% CI: 3.86–15.43), ICU admission (OR = 8.20, 95% CI: 3.90–17.21), and mechanical ventilation (OR = 11.46, 95% CI: 6.33–20.77).

## Discussion

This study demonstrates that blast injuries impose a heavy institutional burden on a Level I trauma center. Although in-hospital mortality was low and non-statistically different between blast and blunt cohorts (1.2% vs. 1.4%, *p* = 0.81), resource utilization across resuscitation, surgery, blood banking, and critical care was multi-fold higher in blast casualties. Blast patients were triaged to the shock-trauma room at more than three times the rate of blunt patients (OR 10.60, 95% CI 8.18–13.75, *p* < 0.0001). This difference was dramatic among the patients grouped as ISS ≥ 16 (97.3% vs. 61.6%, OR 22.73, 95% CI 5.55–93.07, *p* < 0.0001) and persisted even among patients with ISS < 16 (69.1% vs. 16.8%, OR 11.07, 95% CI 8.39–14.60, *p* < 0.0001), indicating that the blast mechanism alone triggers heightened clinical concern regardless of ultimate anatomic injury severity. This aligns with current recommendations that all patients with suspected blast exposure require systematic evaluation for mechanism-specific injuries, including occult pulmonary barotrauma, tympanic membrane rupture, and delayed traumatic brain injury [2, 6, 7]. The high rate of urgent operative intervention (OR 7.49, 95% CI 5.73–9.80, *p* < 0.0001) and the need for three or more operations (OR 12.40, 95% CI 8.59–17.90, *p* < 0.0001) reflect both the immediate severity of blast injuries and the staged nature of their surgical management, encompassing serial debridement, damage control procedures, delayed primary closure, and management of wound complications [8, 9]. MTP was activated in blast patients at more than twelve times the rate of blunt patients (OR 11.68, 95% CI 6.48–21.05, *p* < 0.0001). This reflects the hemorrhagic complexity of explosive injuries: the combination of non-compressible torso hemorrhage, penetrating fragment injuries, solid organ damage, and traumatic amputations creates a high risk of exsanguinating haemorrhage. Mediastinal injury has been identified as the strongest predictor of mortality in mounted blast casualties [10], and early MTP activation has been associated with improved survival, with each minute of delay linked to a measurable increase in mortality risk [11]. Our data reinforce the need for immediate blood product availability and pre-established MTP protocols in centres receiving blast-injured patients. ICU admission rates were more than five times higher in blast patients (OR 5.54, 95% CI 4.37–7.04, *p* < 0.0001). Furthermore, in our propensity-matched cohort, the demand for mechanical ventilation was significantly higher in the blast group (25.9% vs. 4.4%, OR = 7.63, CI 95%: 5.83–9.98, *p* < 0.0001). The mechanical ventilation duration was also significantly longer in the blast patients (11.30 ± 18.50 vs. 4.80 ± 8.20 days, Median: 4.0 vs. 2.0 days, *p* = 0.0012). We do not have documented data on the incidence of primary blast lung injury, or secondary acute lung injury (Acute Respiratory Distress Syndrome, ARDS) in the study groups. However, we assume that this observation of greater demand for mechanical ventilation stems from the various effects of explosion on the lungs. Primary overpressure shockwaves disrupt the alveolar-capillary membrane, causing intra-alveolar hemorrhage, pulmonary contusions, and severe ventilation-perfusion mismatch. Managing blast-induced acute lung injury demands high-intensity critical care strategies including lung-protective low tidal volume ventilation, prone positioning, advanced pulmonary hygiene, and prolonged weaning protocols which directly may account for the nearly six-fold increase in mechanical ventilation rates and elevated ICU utilization observed in this cohort. Furthermore, patients exposed to explosion can develop severe ARDS from toxic gas or smoke inhalation (quaternary effect), up to the stage that veno-venous extracorporeal membrane oxygenation (ECMO) may be necessary as a rescue strategy. A recent meta-analysis demonstrated that ECMO is associated with significantly lower mortality in trauma-induced ARDS (OR 0.29; 95% CI 0.14–0.62, *p* = 0.001) compared to conventional mechanical ventilation, albeit with prolonged ICU stays and substantial resource demands [12]. These data underscore the importance of ECMO capability or established transfer protocols to ECMO-capable centers for institutions receiving blast casualties [13, 14]. Total hospital LOS was significantly longer in blast patients, driven primarily by the severely injured subgroup, while no significant difference was observed among patients with ISS < 16. The extended hospitalization reflects the cumulative effect of staged surgical procedures, prolonged mechanical ventilation, rehabilitation following devastating extremity injuries, and management of wound complications - all hallmarks of blast trauma care [5, 8]. An interesting finding was the lower frequency of major long-bone extremity fractures (AIS ≥ 3) in our blast cohort compared to blunt trauma (43.3% vs. 81.3% and vs. 62.0% in the post PSM blunt group, *p* < 0.0001). In a combat trauma series that presented data from the Iraq and Afghanistan conflicts, extremity injuries predominate, accounting for 51.9% of all combat wounds, with explosive blasts serving as the primary mechanism in 77.9% of cases and severe injuries (AIS ≥ 3) comprising 25.8% of extremity trauma [15]. This incidence is even lower than the one in our series. There is some difference between the presented cohort in the military literature of the mentioned conflicts in Iraq and Afghanistan and our blast cohort. The former focuses heavily on blast mechanism that is inflicted on dismounted foot patrol soldiers exposed to ground-level buried Improvised Explosive Device (IED), resulting in lower extremity traumatic amputations and severe skeletal disruption that predominate due to the direct axial upward shockwaves [16]. In contrast, our civilian/military mixed cohort largely involved open-air detonations, enclosed structure explosions (vehicles or buildings), and indirect rocket/artillery strikes, where primary blast overpressure, secondary fragment impacts, and the tertiary effect of blunt and crush trauma yield far less predictable anatomical injury patterns that do not uniformly target the lower limbs.

A plausible explanation for the gap between blast and blunt trauma is that a substantial proportion of our blunt trauma control population comprises elderly patients presenting with a low-energy isolated femur fracture that are similarly categorized as AIS 3 yet represent a fundamentally different pathophysiological entity than high-energy blast-induced limb trauma. Crucially, despite a lower total skeletal fracture rate, blast casualties suffered extensive soft-tissue devastation, high-energy traumatic amputations, and severe tissue contamination. These complex tissue beds drove an elevated risk of early exsanguination, a tenfold increase in MTP activation (OR = 12.27, 95% CI: 3.24–46.54), and a primary requirement for serial cleansing, surgical debridement and staged wound closure (≥ 3 operations: OR = 11.56, 95% CI: 6.67–20.02). Total hospital LOS and ICU LOS were significantly longer in blast patients. The extended hospitalization reflects the cumulative effect of staged surgical procedures, prolonged mechanical ventilation, rehabilitation following devastating extremity injuries, and management of wound complications - all hallmarks of blast trauma care [5, 8]. A central and clinically important observation in this study is that increased resource utilization in blast patients persisted across both ISS strata, including among patients with ISS < 16 - those generally considered to have minor injuries. Blast patients with ISS < 16 still had significantly higher rates of shock-trauma room activation, urgent surgery, multiple operations, MTP activation, and ICU admission compared to blunt patients in the same ISS stratum (all *p* < 0.0001; see Table 3). This finding supports the concept that mechanism-specific factors - independent of anatomic injury severity as captured by ISS - substantially drive the clinical burden of explosive injuries. Traditional ISS thresholds may also underestimate the true severity of blast trauma due to the saturation effect of injury scoring [17], and an optimal ISS cut-off of 12.5 (rather than 16) has been proposed for predicting mortality and resource requirements in combat trauma [18]. In the present study we found no significant difference in mortality even when the cut-off of ISS 12 was used (*p* = 0.07, not shown in the Results section). Seemingly, the persistence of significant differences across ISS strata argues strongly for mechanism-based clinical protocols that are not contingent on injury severity score alone. In this study mortality was not one of the primary or secondary endpoints, however, this outcome should receive consideration. The mortality rates between blast and blunt patients were similar (1.2% vs. 1.4%, *p* = 0.81), despite lower probability of survival (median 99.28%, IQR 99.01–99.33 vs. 99.42%, IQR 96.75–99.61, *p* = 0.0038) and markedly greater resource demands in the blast cohort. The explanation for this discrepancy may reflect two.

complementary factors. First, the blast patients were mostly healthy males in significantly younger ages (25 vs. 39 years) who have greater physiological reserve - a well-recognized predictor of survival in trauma. The demographic profile of our blast cohort − 97.2% male and a mean age of 25 years - differs markedly from that reported in purely civilian blast series, where mixed-sex populations with older mean ages are typical. For example, in the Beirut Port explosion, the mean age of hospitalized blast casualties was 44.7 years with no substantial male predominance (44.4% were female) [5]. In contrast, the demographic characteristics of our cohort closely mirror those reported in combat trauma registries, where male predominance exceeds 97% and median age ranges from 25 to 29 years [18]. This pattern likely reflects the inclusion of combat-related casualties within our case-mix, and underscores the unique position of our institution at the interface between civilian and military trauma care. Second, our institution’s unique position as a civilian Level I trauma center receiving both civilian and combat-related blast casualties, with blast injuries accounting for 3.4% of admissions - a 10-fold higher proportion than most Western civilian centers [1] - likely contributes to an institutional familiarity with the time-critical and multidisciplinary demands of explosive injuries. Comparison with the literature on other mass casualty incidents shows that our findings mirror experience from major civilian mass-casualty events, such as the Boston Marathon bombing [19], the Beirut Port explosion [5], and victims of suicide bombing incidents [20]. These reports demonstrate that well- organized and trained trauma centers are able to maintain overall survival rates > 95% through mature trauma activation and damage control strategies. However, it is accepted that explosion casualties generate severe surge demands on operating rooms, blood banks, and intensive care units. Together, these factors may explain how a comparable mortality rate in both our study groups was maintained despite the substantially greater clinical burden imposed by blast trauma. That said, the apparent equivalence in mortality should be interpreted with caution, as it may also reflect the net balance of competing variables rather than true equivalence in injury lethality. We therefore emphasize that the primary contribution of this study is not the mortality comparison per se, but the demonstration of a consistently greater resource burden in the blast cohort across both ISS strata.

## Conclusion

Blast trauma imposes a significantly greater institutional burden on Level I trauma centers compared to blunt trauma, despite similar in-hospital mortality rates. Blast-injured patients require substantially higher rates of shock-trauma room activation, urgent surgical intervention, multiple reoperations, MTP activation, ICU admission, and mechanical ventilation, with prolonged hospital and ICU lengths of stay. Crucially, these differences persist across both high and low injury severity strata, indicating that the increased resource demands of blast trauma are driven by the mechanism itself and not solely by anatomic injury severity as measured by ISS.

The comparable mortality between cohorts, achieved in the context of markedly greater resource utilization, suggests that intensive coordinated care can mitigate the inherent lethality of explosive injuries. Level I trauma centers serving populations at risk from combat, terrorism, or industrial explosions should develop mechanism-specific clinical protocols, ensure adequate surge capacity and blood product availability, establish ECMO capability or transfer agreements, and conduct regular training exercises incorporating blast injury scenarios. The structured transfer of knowledge and protocols from military trauma systems to civilian centers represents a critical opportunity to optimize outcomes for this uniquely challenging patient population.

## Limitations & future directions

The retrospective design and reliance on registry data introduce potential ascertainment bias and limit adjustment for confounding variables, including age, sex, and mechanism-specific injury patterns. The application of PSM in this study only partially solves this bias. The absence of multivariate analysis means that the mortality comparison in particular should be interpreted with caution. The single-center design, while providing consistency in care protocols, may limit generalisability to centers with different patient populations or resource profiles. Long-term outcomes, including functional recovery, quality of life, and psychological sequelae such as post-traumatic stress disorder, were not captured. These represent important areas for future investigation. Pure penetrating trauma (e.g., isolated stabs or low/high-velocity gunshot wounds) was intentionally excluded to isolate the unique multi-modal mechanisms of explosions without confounding from isolated knife/bullet trajectories. Blunt trauma was selected as the primary control group because it constitutes > 85% of civilian Level 1 trauma centers volume, representing baseline institutional resource allocation better than that of penetrating trauma. Future multi-center studies should be considered to incorporate a third comparison arm evaluating high-ISS penetrating trauma alongside blast and blunt cohorts.

## Data Availability

The data that support the findings of this study are available from the Israel National Trauma Registry in Gertner institution, but restrictions apply to the availability of these data, which were used under permission for the current study and so are not publicly available. The data are, however, available upon request and with a given permission of Gertner institution.

## Declarations

### Purpose

We hypothesized that blast trauma results in a greater institutional resource burden than blunt trauma.
